# Dr. Ramanan Laxminarayan: antimicrobial resistance: we may be in the darkest hour before the dawn

**DOI:** 10.1017/ash.2023.479

**Published:** 2023-11-06

**Authors:** Ramanan Laxminarayan

**Affiliations:** 1 One Health Trust, Washington, DC, USA; 2 Princeton University, Princeton, NJ, USA

Ramanan Laxminarayan is the Founder and President of the One Health Trust, founded as the Center for Disease Dynamics, Economics & Policy (CDDEP). He is a senior research scholar at Princeton University, an affiliate professor at the University of Washington, a senior associate at the Johns Hopkins Bloomberg School of Public Health, and a visiting professor at the University of Strathclyde in Scotland and the National University of Singapore. Dr. Laxminarayan chairs the board of the Global Antibiotic Research & Development Partnership (GARDP), a global product development partnership created by the World Health Organization, which aims to develop and deliver new treatments for bacterial infections. He is the founder and board chair of HealthCubed, which works to improve access to healthcare and diagnostics worldwide.

Since 1995, Dr. Laxminarayan has worked to improve the understanding of antibiotic resistance as a problem of managing a shared global resource. His work encompasses extensive peer-reviewed research, public outreach, and direct engagement across Asia and Africa through the Global Antibiotic Resistance Partnership. Through his prolific research, active public outreach (including a TED talk that has been viewed over a million times), and sustained policy engagement, he plays a central role in bringing the issue of drug resistance to the attention of leaders and policymakers worldwide and to the United Nations General Assembly in September 2016.

During the Obama Administration, Dr. Laxminarayan served on the U.S. President’s Council of Advisors on Science and Technology’s antimicrobial resistance working group and was a voting member of the U.S. Presidential Advisory Council on Combating Antimicrobial Resistance.

From 2003 to 2004, he served on the National Academy of Science/Institute of Medicine Committee on the Economics of Antimalarial Drugs and subsequently helped create the Affordable Medicines Facility for malaria, a $450 million novel financing mechanism for antimalarials that reduced the cost of antimalarials worldwide. In 2012, Dr. Laxminarayan created the Immunization Technical Support Unit that supports the immunization program of the Ministry of Health and Family Welfare of the Government of India, which is credited with helping introduce four new vaccines and extending vaccination coverage to three million infants who were previously not covered. He was Vice President, Research and Policy at the Public Health Foundation of India between 2011 and 2015, and he led the growth of a research division to over 700 technical and research staff.

Dr. Laxminarayan is a fellow of the American Academy for Advancement of Science and of the Infectious Diseases Society of America and a member of the Council on Foreign Relations. He was named a distinguished alumnus by the Birla Institute of Technology and Science, Pilani in 2019 and by the University of Washington, Department of Economics in 2020. He is a winner of the Ella Pringle medal by the Royal College of Physicians in Edinburgh (Pringle was the first-ever woman elected to the RCPE) and the BP Koirala medal in honor of Nepal’s first democratically elected Prime Minister. Dr. Laxminarayan’s work is widely covered in major media outlets including the New York Times, Washington Post, Associated Press, BBC, Financial Times, CNN, the Economist, LA Times, NBC, NPR, Reuters, *Science*, *Wall Street Journal*, and the *National Journal*. His research includes over 300 books, book chapters, and peer-reviewed papers in leading journals in science, medicine, and economics.

Edited for brevity and clarity.

**You were born in Uganda but studied in the US and work extensively with organizations and governments throughout the world. You’re currently speaking to us from Japan. How did you become this “global citizen” of public health and what does a typical day or week look like for you?**

Well, I am usually either in India at the One Health Trust’s campus in rural Karnataka or in Princeton, New Jersey. Lately I’ve been in Japan as well. Global health takes you to different places, and that’s one of the joys of doing this work. A standard day for me involves working with a wide range of people from diverse backgrounds in different time zones.

**Tell us about your career trajectory. How did you become involved in diverse topics like antimicrobial resistance, malaria, vaccines, COVID, and healthcare diagnostics? What do all your projects have in common in terms of appealing to your personality?**

I started life as an engineer but was passionate about the environment. The first thing I attempted to do after I graduated college was to organize the cleaning of two extremely polluted rivers in the city where I grew up, Chennai in southern India. What I soon found out was that the reason the rivers were polluted with sewage was not because the city didn’t know how to treat its sewage but rather because the poorest residents of the city who lived on the banks of the river had no voice and no ability to influence the city’s priorities. I realized that to solve these social issues I had to acquire some skills beyond engineering.

So that’s why I trained in both economics and epidemiology. I am fascinated with these problems that involve global commons—like pandemics, antimicrobial resistance (AMR), and climate change—that require many people to think beyond their narrow self-interest. For instance, when we overuse antibiotics, others have fewer options to treat infections. And if we pollute the environment, other people don’t get to enjoy the planet as much. A problem like AMR is very challenging because it requires cooperation on different scales, from communities and hospitals, all the way up to nations, cooperating to solve the crisis. AMR is more than just a medical issue in the same way that climate change is more than just an atmospheric science issue. The most interesting problems to solve, whether ozone depletion or infectious diseases, all require multiple disciplines, and that space between disciplines is where I am most at home.

**Tell us about the One Health Trust (formerly CDDEP) and your approach to combatting antimicrobial resistance. How does your work complement that of governments and organizations like the WHO and CDC?**

The One Health Trust was founded to allow researchers at universities to be involved in global, multidisciplinary projects that would typically not be possible or encouraged in discipline-specific university departments. I think the most interesting questions these days lie between disciplines rather than within single disciplines. For that reason, I’ve never seen myself as only an economist, only an epidemiologist, or only an engineer. Also, operating outside of university departments and large institutions including multilaterals enables us to say and do things and convene in ways which governments and other organizations shy away from because they must worry about public perception. So, essentially it gives you more leverage and freedom to look at things across a variety of disciplines.

**In your opinion, what are the three important public health issues of our time, and how are you currently involved in these issues?**

I think the three most critical public health issues of our time are climate change and environmental destruction, pandemics, and antimicrobial resistance. Climate and environmental change has impacted nutrition, water, and influences almost everything to do with public health. The planet will do fine without us but we need this planet to survive. Pandemic risk is also increasing because of the speed of movement of pathogens around the planet, increased exposure to these pathogens particularly in wild habitats, and a large-scale, unprecedented increase in the consumption of animal protein. The third issue, antimicrobial resistance, is occurring primarily because we have built an entire medical system that depends on antibiotics, and if that had not been the case, it would be less of a concern. But given that we absolutely require antibiotics for almost everything in modern medicine, we are on shaky ground. Because it has been a slow-moving “train wreck,” we have failed to recognize that it is, at the end of the day, a pandemic that has been with us for a while.

**What are the top three action items most urgently required to address antimicrobial resistance on a global scale (from individual actions to government and industry actions)?**

The first is to prevent the need for antibiotics, which is perhaps done best through promoting access to clean water and sanitation, vaccines, infection prevention, and antimicrobial stewardship. I think there are tremendous opportunities to prevent infections, and the gap between rich and poor countries is very stark in that respect. We often use antibiotics as a substitute for infection control, water, and sanitation rather than as a corollary to these.

The second would be to focus on not only new drug and vaccine development but also on providing access to these drugs and vaccines. You know there are probably 100,000 lives that could be saved with access to penicillin, because many cases of pneumococcal pneumonia are easily treated with penicillin. Yet that doesn’t happen because penicillin is not a profitable drug to make in many parts of the world and therefore is simply not available.

The pharma industry worldwide is not able to provide access to the cheap, affordable, and effective antibiotics that still work and should be deployed much more widely. The incentives are all in favor of newer antibiotics, which really should not be used and are way too expensive. And sadly, there is not much of an incentive to bring new drugs to market either.

The third agenda, I think, is related to behavior and social norms. We saw during COVID that you can have a great vaccine or guidelines for mask wearing, but if people don’t want to mask or take the vaccine, then you have a problem. It’s an issue of social norms; these are not behaviors that are driven by the individual’s assessment of the pros and cons. Rather, they’re driven by the viewpoint of the social groups to which individuals belong. For instance, individuals in some groups may pretend not to have taken the vaccine even though they’ve had the vaccine or pretend not to have taken the vaccine even though they are vaccinated, just to be consistent with the ideology of the groups to which they belong. I remember when Dr. Francis Collins, the former director of the National Institutes of Health was asked about what he thought his organization could have done differently over his tenure and beyond in tackling a pandemic. He said that perhaps they should have done a little more to understand how people behave because that’s what we really didn’t understand during the COVID pandemic.

**You have a long track record of working with governments, like the Obama Administration, and the Health Ministry of India. Why is this partnership so critical to really making a difference in public health?**

Public health refers to the interests of all citizens and therefore it is something that governments should be invested in. And it’s essential that governments play a leading role. Governments deploy interventions and services on a much broader scale than the private sector and academia. But at the same time, the public sector is not always incentivized to proactively tackle issues. They often prefer tackling problems after they’ve become a serious threat. Public authorities are rarely recognized for preventing pandemics; they are rewarded for responding to pandemics. In a slightly different, although related context, I recall that the former head of the European Space Agency, which is much less known than NASA, once complained that people only heard of his agency when they had a mishap or explosion and not when they were successful. He joked that perhaps they should publicize a few more failures so that people would know that there was a European Space Agency. I think public health agencies feel the same way. They don’t get rewarded for doing their job well. I think it’s our job to work with them to help them see that there are benefits to engaging earlier and to credit them when their work should be appropriately rewarded.

**Your work has been covered by the NYT, BBC, WSJ, CNN, etc. Why is it important to foster a good working relationship with the lay press to push the public health agenda? What could we do better to harness the power of the press to amplify our message more successfully?**

If one is doing work that should inform the view of policymakers, then it is incumbent on academics to get out of their comfort zone and communicate to a broader audience. But it’s not easy. In any given week there are probably about 20,000 papers that have been published in academia, and perhaps 10 make it out into the media in terms of getting some attention from a broader audience.

Sometimes, we academics think it’s the job of somebody else, like the PR office. One must get back to the job we’re trying to accomplish, which is to enable change, and that means communicating research to changemakers. That change doesn’t happen just because one published a great paper. Early in my career, I was a grantee of the Robert Wood Johnson Foundation, which really emphasized this point and really pushed us to communicate our work, whether to Congress or the media, and that early advice made a big impression on me. The world in general—whether in research, sports, or entertainment—is in competition for the attention of others and having media skills gives one an edge.

**Who are your most influential career mentors and why? What do they have in common?**

There have been so many, starting with Gardner Brown who was my PhD advisor. He taught me much about how to think about the world and my place in it. He once told me that the great advantage of academia was that one could think about whatever one chooses to think about, and that flexibility is a tremendous blessing. Simon Levin and Bryan Grenfell at Princeton have consistently supported everything I do, and that’s an important role of a mentor—to just encourage and enable. Nick White taught me about global health, drug resistance, malaria, and tropical infectious diseases. David Heymann was influential in my understanding of vaccines and the outsize role they will continue to play in the future. Dame Sally Davies inspired me to think big about what we could be doing to solve the problem of antimicrobial resistance. Sir George Alleyne, a former Director General of PAHO and the founding chair of the One Health Trust board, encouraged me to always think about work-life balance and to actively communicate my work, if not to the media at least to other scientists. The work of Rosanna Peeling on diagnostics has been influential in my understanding of the fact that technology is the easiest part of diagnostics, and the incentives and economics are the much more difficult part. There have been many more mentors in my life—too many to name here. At the end of the day, everyone is the sum of all the mentors they have met, and if one is lucky, as I have been, there will be more than a few.

**Our readership is comprised primarily of hospital- or clinic-based professionals in infection prevention and antimicrobial stewardship. How can they get involved with the fight against AMR, on a larger scale, outside the hospital or clinic?**

There is so much to be done. Due to COVID, I think it’s only now that the public appreciates what infection control professionals do within the hospital. People now understand that infections spread easily, and prevention is an important part of watching out for someone else’s well-being as well as one’s own well-being. But I think that the message must be more broad and nuanced at the same time. We’ve had this metaphor of the “war on microbes,” which is a general idea that all microbes are bad, when most microbes are either benign or good for us and it’s only a small handful that are a problem. I think this appreciation of the microbial world should be communicated by the people who deal with it on a day-to-day basis. It would help not to promote disinfectants to the public that kill 99.9 9% of pathogens, which is all driven by an irrational fear. Now that we’ve captured the public’s attention about microbes, it’s time for us to deliver a more nuanced message. Your readership could also think about how to engage globally in settings where infection prevention could be so much better.

**During your global travels, which books, articles, or podcasts are you reading or listening to and which do you recommend for our readers?**

I listen religiously to the podcast put out by the One Health Trust weekly called “One World, One Health,” which is available on Apple Music and Spotify. A recent episode covered how traditional societies did a very good job dealing with COVID because they reverted to methods they developed dealing with past pandemics and fared better. Apart from that, I must confess to being an NPR junkie. When I’m in the U.S. on my morning drive, I listen to “Fresh Air,” or “Morning Edition,” which is one of my favorites. Since the One Health Trust is building an academic retreat, offices, and garden near Bangalore, India, my recent books have either been about architecture or Japanese gardens. For architecture, I am a big fan of Geoffrey Bawa—the renowned Sri Lankan architect and read anything about his work. For anyone interested in the latter—I can recommend—Niwaki: Pruning, training, and shaping trees the Japanese Way which is what I am on right now.

**Finally, do you have any parting message for our readers?**

Sometimes, we treat antimicrobial resistance as an evergreen problem and one we can’t do much about. At times, it’s an issue some think can only be solved with new antibiotics. I don’t think that’s the case. Lots can be done to conserve the antibiotic effectiveness we have currently. And meaningful change is possible. The United States only started acting with purpose on AMR in 2015, and the results are evident. Other countries, mostly in Europe, have shown that progress is possible. I am optimistic that 20 years from now we will have addressed this in a significant way through a combination of tools, and we’ll look back and say that there was a period when AMR was bad, but now things are far better. I think we’ve seen this kind of despair followed by progress with HIV, malaria, and other infectious diseases, including with COVID. But the darkest hour comes before the dawn. I hope we are in that darkest hour with AMR right now and that dawn is not far away.

